# Measuring Shared Electrons in Extended Molecular Systems: Covalent Bonds from Plane-Wave Representation of Wave Function

**DOI:** 10.3390/molecules26134044

**Published:** 2021-07-01

**Authors:** Giovanni La Penna, Davide Tiana, Paolo Giannozzi

**Affiliations:** 1 Institute of Chemistry of Organometallics Compounds (ICCOM), National Research Council (CNR), via Madonna Del Piano 10, I-50019 Sesto Fiorentino, Italy; giovanni.lapenna@cnr.it; 2 National Institute of Nuclear Physics (INFN), Section of Roma-Tor Vergata, via Della Ricerca Scientifica 1, I-00133 Roma, Italy; 3 School of Chemistry, University College Cork, T12 YN60 Cork, Ireland; davide.tiana@ucc.ie; 4 Department of Mathematics, Computer Science, and Physics, University of Udine, Via Delle Scienze 206, I-33100 Udine, Italy; 5 Istituto Officina dei Materiali (IOM), National Research Council (CNR), SISSA-ISAS, I-34136 Trieste, Italy

**Keywords:** delocalization index, bond order, density-functional theory, high-performance computing

## Abstract

In the study of materials and macromolecules by first-principle methods, the bond order is a useful tool to represent molecules, bulk materials and interfaces in terms of simple chemical concepts. Despite the availability of several methods to compute the bond order, most applications have been limited to small systems because a high spatial resolution of the wave function and an all-electron representation of the electron density are typically required. Both limitations are critical for large-scale atomistic calculations, even within approximate density-functional theory (DFT) approaches. In this work, we describe our methodology to quickly compute delocalization indices for all atomic pairs, while keeping the same representation of the wave function used in most compute-intensive DFT calculations on high-performance computing equipment. We describe our implementation into a post-processing tool, designed to work with Quantum ESPRESSO, a popular open-source DFT package. In this way, we recover a description in terms of covalent bonds from a representation of wave function containing no explicit information about atomic types and positions.

## 1. Introduction

The calculation of the ground-state electron density in an extended atomic system is important in order to understand and guide the design of properties and functions of materials and molecular assemblies. A method providing an excellent compromise between computational cost and accuracy of the results is density-functional theory (DFT), in which the electron density is represented in terms of effective one-electron Kohn–Sham (KS) states [1]. DFT equations are frequently solved by expanding KS states into plane waves (PW), embedded in the periodic representation of the sample of atoms. Atoms are represented by means of the respective frozen atomic cores, via the pseudo-potential approach, and the frozen cores act on a completely delocalized representation of valence electrons. Such representation is natural for periodic systems such as crystalline solids, but can be applied to liquid and disordered materials as well using large super-cells and periodic boundary conditions, as in first-principle molecular dynamics simulations [2]. With these approximations, the calculation of the ground-state electron density can be performed on high-performance computers for systems in the order of a thousand atoms. This corresponds to a super-cell size of about 2 nm side, affordable to first-principle molecular dynamics.

The calculation of the various quantities describing the covalent bond order is a very useful tool for analyzing first-principle results in terms of simple chemical concepts. The importance of routine calculation of such quantities has been recently stressed [3]. In the following, we will focus on the delocalization indices (DI) [4].

In DFT calculations using PWs, chemical bonds cannot be easily identified: in fact, the PW basis set used to represent the electron density and KS states is delocalized and does not explicitly depend upon atomic coordinates. As a consequence, the calculation of DIs is traditionally based on localized, atom-centered basis sets and all-electron wave functions. While highly accurate, such an approach is limited to isolated and relatively small molecules. Interesting alternative analyses of wave function and related properties are based on fuzzy atoms [5,6], ring current [7], and on Wannier functions [8,9]. These techniques, however, are limited to aromaticity or suffer from some arbitrariness, and require demanding additional calculations to those leading to the original wave function.

A few calculations of the bond order based on DFT calculations and PWs are known [9,10]. Both applications are limited to a few tens of atoms because they require post-processing steps on high-resolution wave function and charge density. In the first case [10], projector-augmented waves are used. For the largest-size application shown in this paper, a real-space representation of the electron density requires a grid of about 216 points per side, close to the limits of popular analysis codes like DGrid [11] or Critic [12]. In the second case [9], the limitation due to the large size of the real-space grid is circumvented by the additional use of maximally localized Wannier functions to optimize the compute-intensive grid-based integration.

In this work, we describe a straight application that circumvents the numerical problems described above with no change in electron density representation. Our work extends the implementation of the method for computing DIs, previously applied to small isolated molecules [13], for all atomic pairs in an extended system. We provide the related software as a post-processing tool for usage within the Quantum ESPRESSO (QE) distribution [14,15]: an integrated suite of open-source computer codes for electronic-structure calculations and materials modeling at the nanoscale. As an example of application, we consider models of CO-coated Pt nano-clusters. Our implementation allows to quickly compute DIs in super-cells containing up to 120 heavy atoms. The post-processing tools added to QE measure the number of electrons shared between any pair of atoms, directly from the ground state wave function represented on the basis of plane-waves and pseudo-potentials. Atoms are assigned to atomic basins, which are regions of space including each atomic core. Atomic basins are not uniquely identified, but the quantum theory of atoms in molecules proposed by R. Bader [16,17] showed that a method based on the electron density gradient is less sensitive to the wave function representation (basis-set) than other types of assignments. We therefore used the numerical description of atomic basins as it is implemented in the code developed by the group of G. Henkelman [18,19].

This work aims at describing the implementation of the method, while a deeper analysis of DIs and their possible correlation with stability conditions and electronic properties will be the subject of further studies.

## 2. Computing Electron Sharing

### 2.1. Delocalization Indices

The equation for the delocalization index δ between each atom pair is well known [4] and involves the integral, in the space spanned by each of the atomic basins, of the overlap between all pairs of different KS states:

(1) $$\delta \left(A,B\right)=2\sum_{i,j}\left[n_{i,\alpha }n_{j,\alpha }+n_{i,\beta }n_{j,\beta }\right]S_{i,j}\left(A\right)S_{i,j}\left(B\right)\;,$$

where *i* and *j* run over all the occupied KS states, $S_{i,j}\left(A\right)$ are overlap integrals of the *i*-th and *j*-th KS states, integrated within the region of space identified as the basin of atom *A*. The numbers $n_{i,\alpha }$ are the occupations of each spin orbital. The latter occupations can be fractional for metallic systems, or for molecular systems with a degenerate ground state. The *localization* indices λ are defined as

(2) $$\lambda \left(A\right)=\sum_{i,j}\left[n_{i,\alpha }n_{j,\alpha }+n_{i,\beta }n_{j,\beta }\right]S_{i,j}{\left(A\right)}^{2}.$$

Equations (1) and (2) hold for general spin unrestricted single-determinant wave function (Hartree–Fock level), like it is the case in Kohn–Sham spin collinear approximation. In our applications, we consider only spin-restricted cases. The generalization to spin-unrestricted cases is straightforward and was actually used in the first calculation of DIs of ref. [13], for the CO dissociation. The crucial quantities for the calculation of DIs are the overlap integrals $S_{i,j}$:

(3) $$S_{i,j}\left(\Omega \right)=\int_{\Omega }\psi_{i}^{*}\left(\vec{r}\right)\psi_{j}\left(\vec{r}\right)d\vec{r}\;,$$

where the $\psi_{i}$’s are the KS states and Ω indicates the integration basin for the chosen atom. KS states in periodic systems are in general Bloch states and are characterized by a wave vector $\vec{k}$ in addition to the band index. For large super-cells, however, we may limit them to the Γ ($\vec{k}=0$) point only. In such a case, the $\psi_{i}\left(\vec{r}\right)$ functions are real and the complex conjugate can be dropped. In the following, we postpone the identification of the basins to Section 4.2 and Section 4.3 and focus on the calculation of the integrand in Equation (3).

### 2.2. Ultra-Soft Pseudo-Potentials

Many DFT calculations based on PWs employ ultra-soft pseudo-potentials for their computational efficiency [20]. In the ultra-soft pseudo-potential scheme, the charge density is given by the following expression:

(4) $$\rho \left(\vec{r}\right)=\sum_{i}\left(|\psi_{i}\left(\vec{r}\right){|}^{2}+\sum_{l,m}\sum_{\mu }q_{lm,\mu }\left(\vec{r}-{\vec{R}}_{\mu }\right)\langle \psi_{i}|\beta_{l,\mu }\rangle \langle \beta_{m,\mu }|\psi_{i}\rangle \right)$$

where the $\psi_{i}$’s are represented as linear combinations of PWs, 〈…〉 is the scalar product in Dirac notation, ${\vec{R}}_{\mu }$ is the atomic position for the μ-th atom, the $q_{lm,\mu }$ functions are *augmentation charges*, and the $\beta_{l,\mu }$ functions (*projectors*) define the non-local part of the pseudo-potential. KS states are subject to a generalized normalization condition of the form:

(5) $$\langle \psi_{i}|\hat{O}|\psi_{j}\rangle \equiv \int \psi_{i}^{*}\left(\vec{r}\right)\psi_{j}\left(\vec{r}\right)d\vec{r}+\sum_{l,m}\sum_{\mu }Q_{lm,\mu }\langle \psi_{i}|\beta_{l,\mu }\rangle \langle \beta_{m,\mu }|\psi_{j}\rangle =\delta_{ij},$$

where

(6) $$Q_{lm,\mu }=\int q_{lm,\mu }\left(\vec{r}\right)d\vec{r}.$$

It is important to notice both augmentation charges $q_{lm,\mu }$ and projectors $\beta_{l,\mu }$ are short-range functions, centered around atom μ at site ${\vec{R}}_{\mu }$.

Overlap integrals $S_{i,j}$ in the ultra-soft pseudo-potential framework can be recast under the form:

(7) $$S_{i,j}\left(\Omega \right)=\int_{\Omega }\left(\psi_{i}^{*}\left(\vec{r}\right)\psi_{j}\left(\vec{r}\right)+\rho_{i,j}^{US}\left(\vec{r}\right)\right)d\vec{r}\;,$$

where the augmentation contribution is written as

(8) $$\rho_{i,j}^{US}\left(\vec{r}\right)=\sum_{l,m}\sum_{\mu }q_{lm,\mu }\left(\vec{r}-{\vec{R}}_{\mu }\right)\langle \psi_{i}|\beta_{l,\mu }\rangle \langle \beta_{m,\mu }|\psi_{j}\rangle .$$

In practice, plane-wave components $\psi_{i}\left(\vec{G}\right)$ are computed up to a suitable kinetic energy cut-off $E_{w}$. KS states and their products $\psi_{i}^{*}\psi_{j}$ are quickly and easily computed on a discrete real-space grid of points via Fast Fourier transform (FFT). The integral becomes a sum over grid points belonging to the basin of each atom. We remark that it is customary and convenient to use a denser real-space grid for the augmentation terms than the one used to compute the products of KS orbitals. The latter must contain PWs up to a larger kinetic energy cut-off, $E_{c}=4E_{w}$, while the “dense” grid contains PWs up to an even larger cut-off, typically $E_{d}=8÷12E_{w}$.

The evaluation of the augmentation term, Equation (8), is the time-consuming step. The typical way (implemented in QE) to deal with augmentation charges is to compute them in $\vec{G}$-space:

(9) $$\rho_{i,j}^{US}\left(\vec{r}\right)=\sum_{\vec{G}}{\tilde{\rho }}_{i,j}^{US}\left(\vec{G}\right)e^{i\vec{G}\cdot \vec{r}}.$$

$\vec{G}$-space components are in turn written as

(10) $${\tilde{\rho }}_{i,j}^{US}\left(\vec{G}\right)=\sum_{l,m}\sum_{\mu }q_{lm,\mu }\left(\vec{G}\right)e^{-i\vec{G}\cdot {\vec{R}}_{\mu }}\langle \psi_{i}|\beta_{l,\mu }\rangle \langle \beta_{m,\mu }|\psi_{j}\rangle .$$

The calculation of Equation (10) is straightforward: the scalar product between β functions and ψ KS states in the PW basis set reduces to a matrix–matrix multiplication, while $q_{lm,\mu }\left(\vec{G}\right)$ and $\beta_{l,\mu }\left(\vec{G}\right)$ are atomic functions in Fourier space and are easily computed. ${\tilde{\rho }}_{i,j}^{US}\left(\vec{G}\right)$ is then transformed back to real space (on the dense grid) using inverse FFT and added to Equation (7).

This algorithm was used in [13] and is mathematically exact for a given PW basis set, but it is also computationally heavy. The number of needed floating-point operations is $O(M^{2}n_{p}N_{at}N_{PW})$, where *M* is the number of KS states, $n_{p}$ the number of projectors per atoms, $N_{at}$ the number of atoms, and $N_{PW}$ the number of plane waves. Apart from $n_{p}$, a number ∼O(10) depending upon the atomic species, all other factors grow linearly with the size of the super-cell. The computational workload thus grows as the fourth power of the super-cell size. For super-cells containing more than a few tens of atoms, the calculation becomes much heavier than the solution of DFT equations.

### 2.3. Faster Algorithm

In order to obtain a faster algorithm, one needs to exploit the short-range character of augmentation charges and of projectors in real space. By computing Equation (7) in real space, instead of going through reciprocal space, one might gain a rather large factor: the ratio between the total number of grid points and the number of grid points for which the augmentation charges are nonzero. While actually available in QE, such an algorithm is rather complex and not well suited for parallel execution.

Here, we present an even simpler algorithm that increases the speed of the calculation while exploiting existing parallelization schemes (see Section 4.4). The price to pay is that the calculation of Equation (7) is no longer exact, but the results are still very close to exact ones, as shown in the reported application (see Section 3).

We first compute $\hat{O}\psi_{i}$, where $\hat{O}$ is defined in Equation (5), and bring it to real space using FFT. Both operations are already present in QE and can be easily re-used. We then compute

(11) $${\tilde{S}}_{i,j}\left(\Omega \right)\equiv \int_{\Omega }\psi_{i}^{*}\left(\vec{r}\right)(\hat{O}\psi_{j})\left(\vec{r}\right)d\vec{r}.$$

Such quantity differs from overlap integrals $S_{i,j}\left(\Omega \right)$ as defined in Equation (7). In fact,

(12) $$\begin{array}{ccc} {\tilde{S}}_{i,j} & = & \int_{\Omega }\left(\psi_{i}^{*}\left(\vec{r}\right)\psi_{j}\left(\vec{r}\right)+\sum_{lm,\mu }\psi_{i}^{*}\left(\vec{r}\right)\beta_{l,\mu }\left(\vec{r}-{\vec{R}}_{\mu }\right)Q_{lm,\mu }\langle \beta_{m,\mu }|\psi_{j}\rangle \right)d\vec{r} \end{array}$$

(13) $$\begin{array}{ccc} & \neq & \int_{\Omega }\left(\psi_{i}^{*}\left(\vec{r}\right)\psi_{j}\left(\vec{r}\right)+\sum_{lm,\mu }q_{lm}\left(\vec{r}-{\vec{R}}_{\mu }\right)\langle \psi_{i}|\beta_{l,\mu }\rangle \langle \beta_{m,\mu }|\psi_{j}\rangle \right)d\vec{r}. \end{array}$$

Since, however, both $q(\vec{r}-{\vec{R}}_{\mu })$ and $\beta (\vec{r}-{\vec{R}}_{\mu })$ are short-range and centered around an atom, the integral over the atomic basin Ω includes only the core region of the atom in Ω. Under the hypothesis that the core region is completely included into the atomic basin, and remembering Equation (6), one realizes from Equations (12) and (13) that ${\tilde{S}}_{i,j}≃S_{i,j}$.

The calculation of DIs with the algorithm sketched above is much faster and easier to code than the exact calculation of the previous section. The number of needed floating-point operations is $O(Mn_{p}N_{at}N_{PW})$ for $\hat{O}\psi_{i}$, $O(MN_{PW}\log N_{PW})$ for the FFTs, $O(M^{2}N_{PW})$ for Equation (11), and grows no faster than the cube of the super-cell size.

## 3. Applications to an Extended System with Unusual Bonds

We choose a paradigmatic extended system as a test for applying the DI equation. The system has been chosen in the frame of CO-metal clusters, since they present different types of bonds shared by isolated clusters and extended, up to periodic, systems [21,22]. We built models of a CO-isolated Pt nano-clusters, composed by a number of planar Pt_3_(CO)_6_ clusters stacked in the direction of the Pt_3_ plane of each cluster. The general molecular formula of these clusters is [Pt_3_(CO)_6_]_n_^2−^ (*n* = 1–10), where *n* is the number of layers, containing a single [Pt_3_(CO)_6_] unit, that the cluster consists of. These individual units stack on top of one another and arrange into a trigonal prismatic fashion along a pseudo-C${}_{3}$ axis. What is observed with increasing *n* is a lengthening molecular wire of repeating molecular units. It has been found that at low nuclearity, n⩽4, the cluster always crystallizes adopting ionic 0-D packings, with the anions and cations separated by normal van der Waals contacts. On the other hand, for n⩾5, the oligomers tend to self-assemble and can form infinite nano-wires [23,24,25]. For this reason, we modeled the series of compounds [Pt_3_(CO)_6_]_n_^2−^ with n=1,2,4,8. The n=1 case represents the monomer; the n=2,4 cases represent isolated oligomers; the n=8 case represents the infinite nano-wire. The compounds have been minimized in a routinely used DFT model, starting with a super-cell of 2 nm × 2 nm × 2.56 nm, with variable $L_{z}$ in the latter case (see Section 4.1). We computed the DIs for all atom pairs in the series.

**Figure 1:**
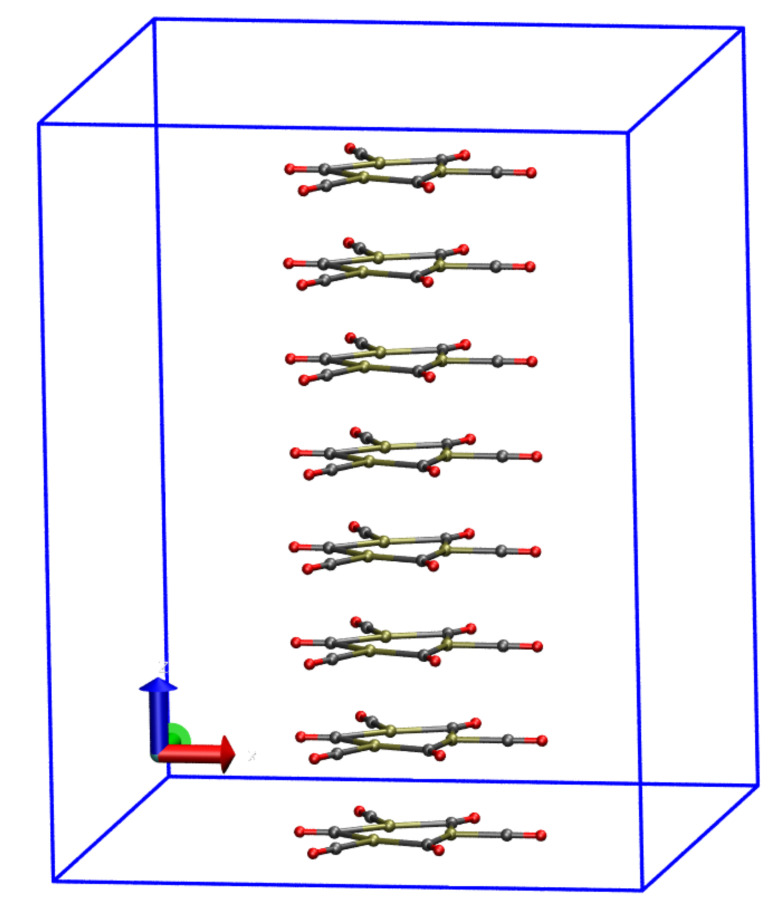
Structure of infinite nano-wire [Pt_3_(CO)_6_]_8_^2−^ in a periodic cell, drawn in blue. The energy is minimized with no counterions and the total charge q=−2. The nano-wire direction is along the *z* axis (blue). Pt, C and O atoms are represented as ochre, gray and red spheres, respectively. Atomic and bond radii are arbitrary. Bonds are drawn when the distance between atoms is shorter than 2.1 Å. The VMD program [26] is used for all molecular drawings.

Systems are composed by 15, 30, 60, and 120 atoms, respectively, with 46, 91, 181, and 363 filled KS states, respectively. The number of points in the real-space representation of density and KS states is 192 × 192 × 243. All calculations were performed with QE v.6.6. The minimal energy configuration obtained for the infinite nano-wire is displayed in Figure 1.

**Figure 2:**
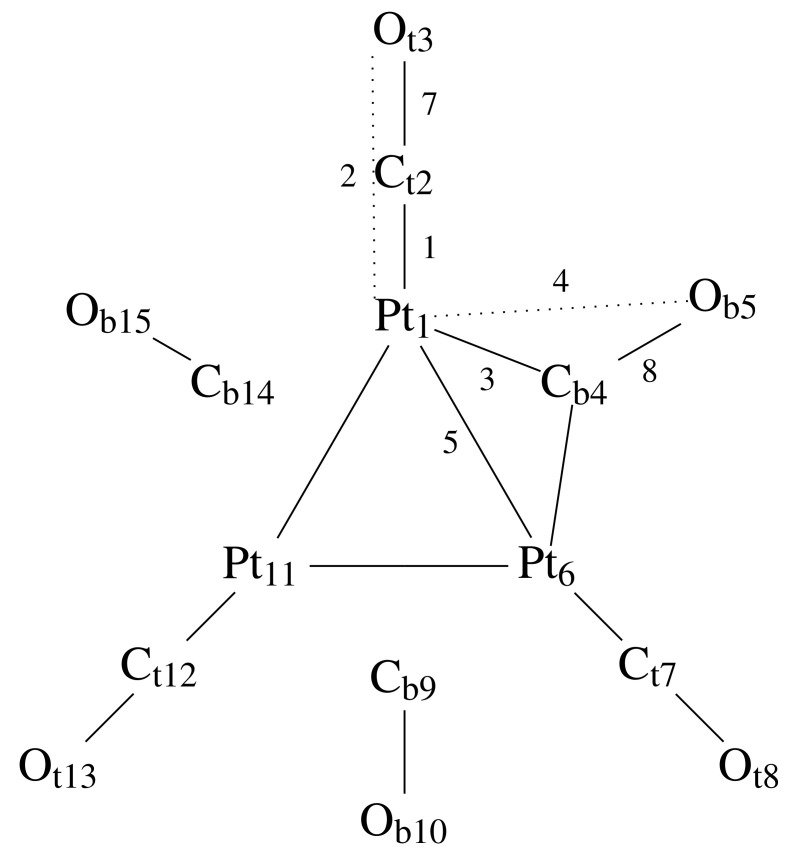
Atom indices within each Pt_3_(CO)_6_ stack, used throughout the description of DIs. Indices of other stacks in dimers and in the nano-wire are incremented by 15, the number of atoms in each stack. Pair type 6 involve Pt atoms of different Pt_3_ stacks.

The values of DI for the monomer, the isolated (n= 2, 4) oligomers and for the infinite (n= 8) nano-wire are reported in Table 1 for the pairs of atoms that are shared among all monomers and for the additional Pt–Pt pairs where Pt are the closest atoms of different monomers. The values are averaged over the equivalent bonds, and atoms in each bond are labeled, for the monomer, as in Figure 2. According to the analysis of PW-based calculation of DI reported in the previous application to simple molecules [13], the error on DI, with this type of approximations, is 0.1 units. Because of this estimated accuracy, we do not report DIs smaller than 0.1 in all of our applications described below. For instance, in the monomer, the DI for atom pairs that are close in space, like Ct2–Cb4 in Figure 2 (3.0 ÷ 3.1 Å), is 0.056 ± 0.002. Other pairs, like Ot3–Ob5 have DIs smaller than 0.05. The main message of Table 1 is that 3 covalent bonds appear between Pt atoms belonging to different stacks (Pt1-Pt16), with minimal change in the covalent bond distribution among each stack involved in the interaction between Pt_3_ planes. The availability of electrons shared between different planes is due to the removal of electrons when planes are stacked one over each other. The major change within each assembled monomer is the decrease of DI for Pt–Ct pairs, from 1.5 in the isolated monomer to 1.3 in the nano-wire. This point will be discussed in more detail below.

As for computational resources and performance: with the original algorithm of Section 2.2, the calculation of DIs for the infinite nano-wire model (120 atoms) takes 37 h of wall-time running over 144 nodes with 3 tasks per node (because of memory limits). The total number of tasks is 432, and is matched in this particular case by the number of KS states (359 states with occupation 2, 4 states with fractional occupation, 69 empty states), allowing an ideal distribution of one state per task. The new algorithm presented in Section 2.3 for the same model takes only 43 s of wall-time running over 16 nodes with 27 tasks per node. The total number of parallel tasks is therefore the same as in the the original algorithm, but more tasks can share the same memory on each node. The performance of the new algorithm is thus much better and may allow an almost real-time on-the-fly calculation.

We notice that in this form the calculation of DIs scales nearly linearly with the number of computational tasks (see Section 4.4). The high number of tasks that is required is about 5–10 times that required for the calculation of ground state wave function in about the same real time. For example, the calculation of the ground state wave function for the infinite nano-wire (120 atoms) takes 260 s of wall-time using 48 tasks running over one modern many-cores node. The calculation of DIs takes 1/5 of the wall-time using about 10 times the tasks. Therefore, the post-processing of wave function on modern high-performance computing architectures is affordable with a moderate effort, but avoiding either large movement of data and changes in the wave function representation.

**Figure 3:**
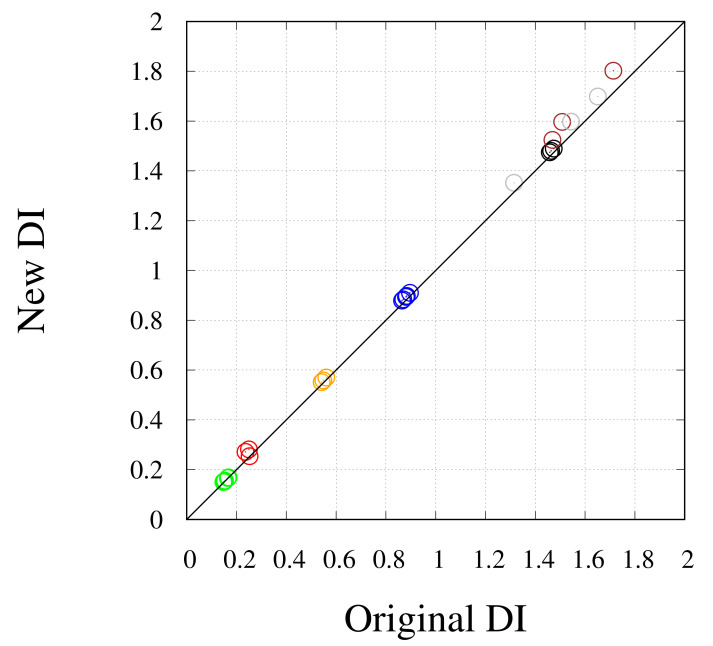
Comparison between DI in the monomer computed with the original (*x* axis) and the new algorithm (*y* axis). Different colors are used for atom pairs indicated in Figure 2: black—Pt1-Ct (pair type 1); red—Pt1-Ot (pair type 2); blue—Pt1-Cb (pair type 3); green—Pt1-Ob (pair type 4); orange—Pt1-Pt1 (pair type 5); brown—Ct-Ot (pair type 7); gray—Cb-Ob (pair type 8).

The price paid for the greater computational efficiency of the new algorithm is shown in Figure 3 for the monomer. The DI values calculated with the new algorithm (*y* axis) are compared to those obtained with the original algorithm (*x* axis). Different colors identify different groups of theoretically equivalent bonds, according to the scheme in Figure 2. The deviation between the two sets of results is within the DI error (0.1). The largest error is shown in the dispersion of DIs corresponding to bonds that are in theory identical by symmetry, irrespective of the algorithm used. This is particularly evident for pairs Ct–Ot and Cb–Ob, that change by 0.4 units (gray and brown circles). This unexpected variation is concentrated at the periphery of the cluster. The reason of this deviation is the low definition of atomic basins in the region of space where the electron density is low and flat, i.e., where there are no atoms. This region can be, in theory, assigned to an additional vacuum basin, but such correction will be included in a further study. A better understanding of this issue is discussed below for dimers.

### 3.1. Dimers

**Figure 4:**
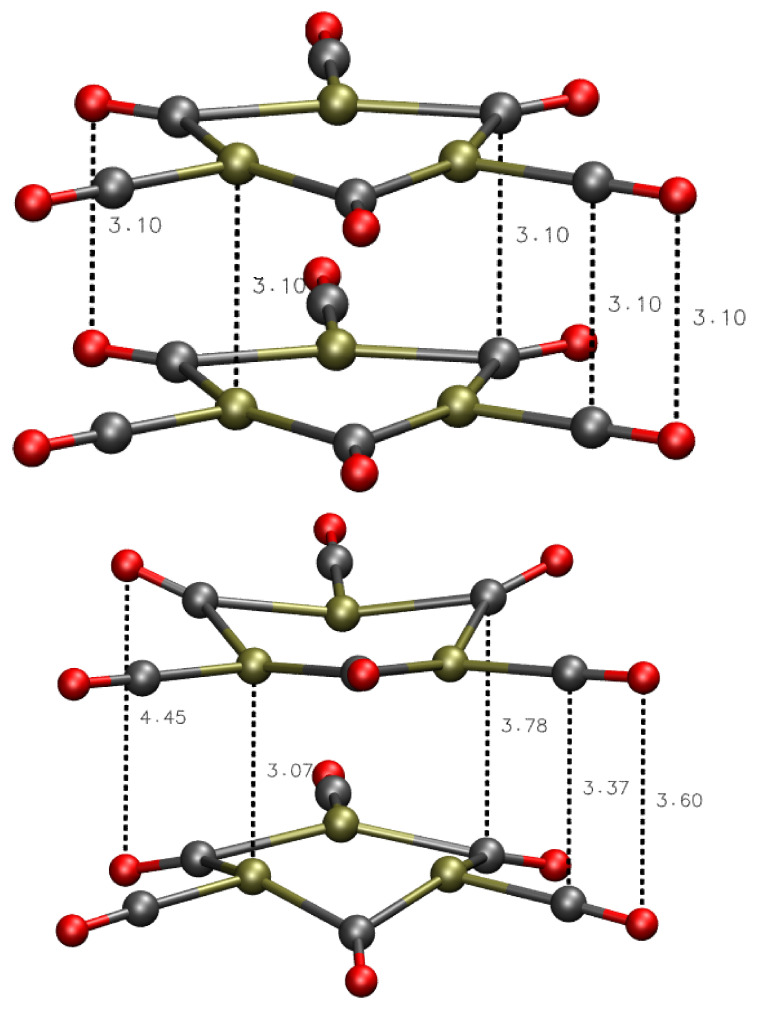
Geometry of dimer with charge q=−2 before (**top**) and after energy (**bottom**) minimization.

We performed calculation of DIs and of charge integrated within the atomic basins connected by DIs, for the same configuration of the dimer obtained by energy minimization with total charge q=−2. Assuming that q=−2 is the optimal charge to stabilize the monomer, the approach between two monomers keeping their respective charge is destabilized by the electrostatic repulsion. Therefore, the oxidation of the dimer upon the assembling process is required to reduce repulsion while keeping enough electrons to be possibly shared within each assembled monomer as in the isolated monomer. On the other hand, the oxidation can perturb the stability of each monomer because electrons can be partially removed by covalent bonds that are essential to seal the monomeric cluster. These bonds are Pt–Pt bonds, but also the C-O bonds that keep the isolating layer made of CO molecules. As a compromise between these effects, the ideal electron removal should not perturb the distribution of electrons compared to the monomer. By observing the change in molecular geometry upon energy minimization (Figure 4), it can be noticed that O terminal atoms (Ot and Ob) increase the relative distance when the O atoms of O–O pairs belong to different facing monomer. Conversely, Pt atoms become closer, strengthening the covalent nature of a little Pt_6_ cluster isolated by CO bound molecules. The latter CO ligands, being highly polarized, tend to repel each other while keeping the Pt cluster isolated.

**Figure 5:**
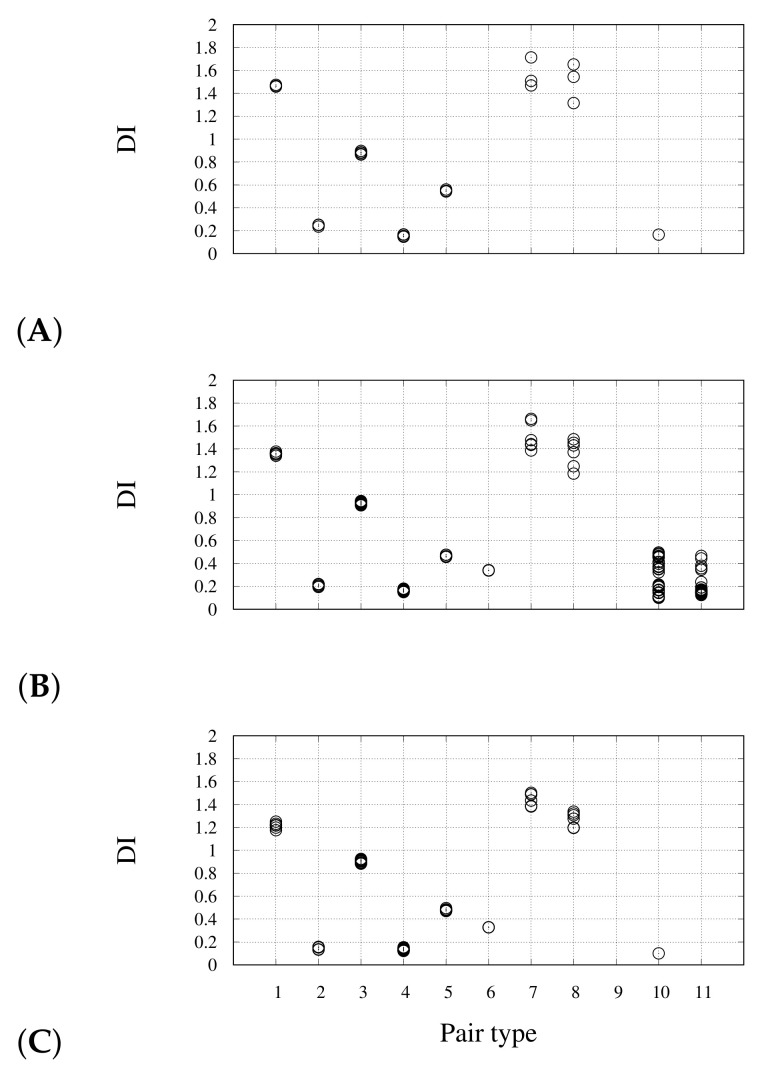
DI (*y* axis) for different atom pairs (see list below and Figure 2) for the isolated monomer (**A**) and for the dimer (**B**), both with total charge q=−2. Panel (**C**) is with the addition of Mg far from molecule (total charge zero). List of atom pairs: 1—Pt1-Ct; 2—Pt1-Ot; 3—Pt1-Cb; 4—Pt1-Ob; 5—Pt1-Pt1; 6—Pt1-Pt16; 7—Ct-Ot; 8—Cb-Ob; 10—different from above, but intra-monomer; 11—as in 10, but inter-monomer.

The comparison of DIs of isolated monomers and of assembled dimers is displayed in Figure 5, where the top panel displays the values for the isolated monomer with charge q=−2. The bond types 10 and 11 are long-range pairs intra-monomer and inter-monomer, respectively. These types of bonds become significantly populated when the total charge of dimer achieves the value q=−2 (B). The recovery of values of DI(Pt–Ct), DI(Pt–Cb), and DI(Pt–Ob) corresponding to the monomer when the negative charge of dimer becomes −2 (panel B) from −4 (data not shown) indicates that bonds around Pt are restored as in the monomer, with no significant change of DI(Pt1–Pt16), thus showing a change of the local Pt environment compared to the isolated monomer. Therefore, we argue that the 2-electron oxidation of the dimer (panel B) represents a state with intra-monomer bonds similar to the isolated monomer, with part of excess electrons shared by Pt–Pt bonds connecting different facing monomers, and the remind of excess electrons pushed as far as possible away from the molecule. The large values of DIs for pairs of type 10 (intra-monomer, up to 1.2) when charge is −2 (panel B) indicates that there are no ways to share excess electrons away from monomers (Pt1-Pt16 and bond types 11). This is an indication that for small oligomers when the total charge is negative (−2) electron density is compressed in separated stacks, with no relaxation of electron-electron repulsion. The addition of a positive hole like a Mg atom, keeping the total charge zero (panel C) shows that the consequent change of electron density gradient allows a better definition of the region where the excess of electrons is spread. The many DIs of types 10 and 11 disappear and no electron sharing between CO and Mg is measured.

### 3.2. Nano-Wire

**Figure 6:**
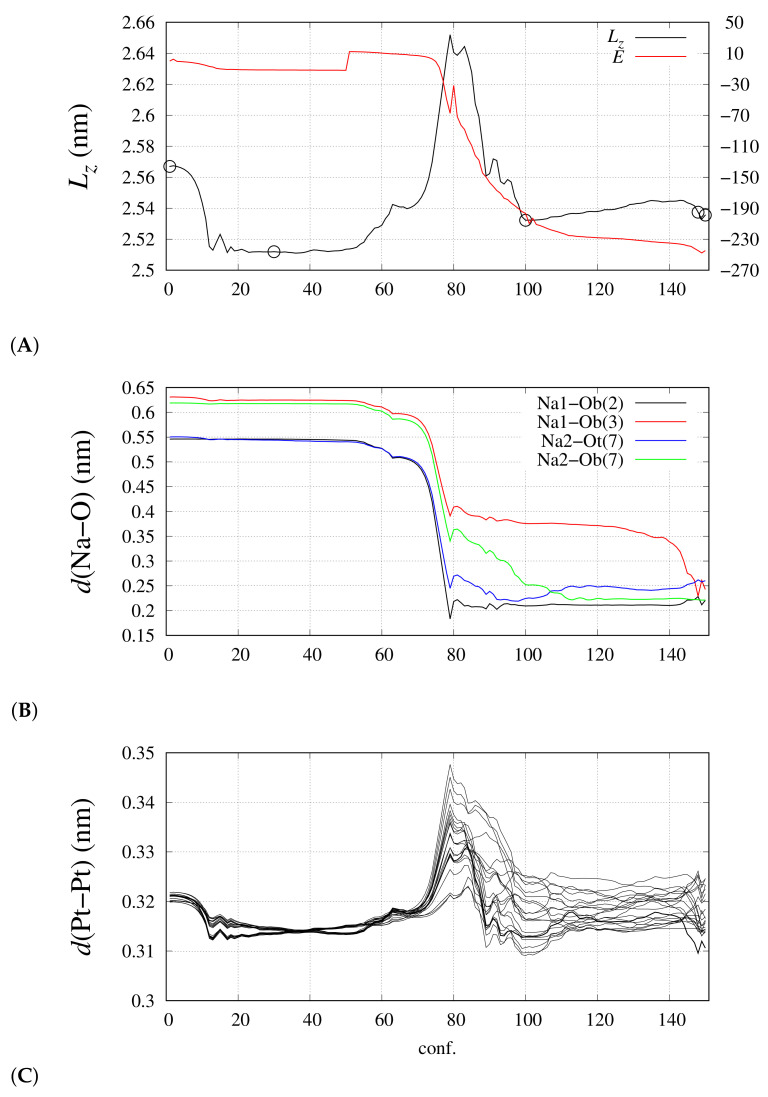
Evolution of geometrical parameters of the nano-wire along with the variable-cell energy minimization. The first 50 configurations are obtained with Na atoms fixed in space. Panel (**A**)—cell length along the *z* direction ($L_{z}$, left axis) and total energy (*E*, right axis, zero is for the initial value); circles emphasize the configurations analyzed in Figure 8; panel (**B**)—distances between the two Na atoms and the closest O atoms of the nano-wire (the monomer number is indicated within brackets); panel (**C**)—inter-monomer shortest Pt distances.

**Figure 7:**
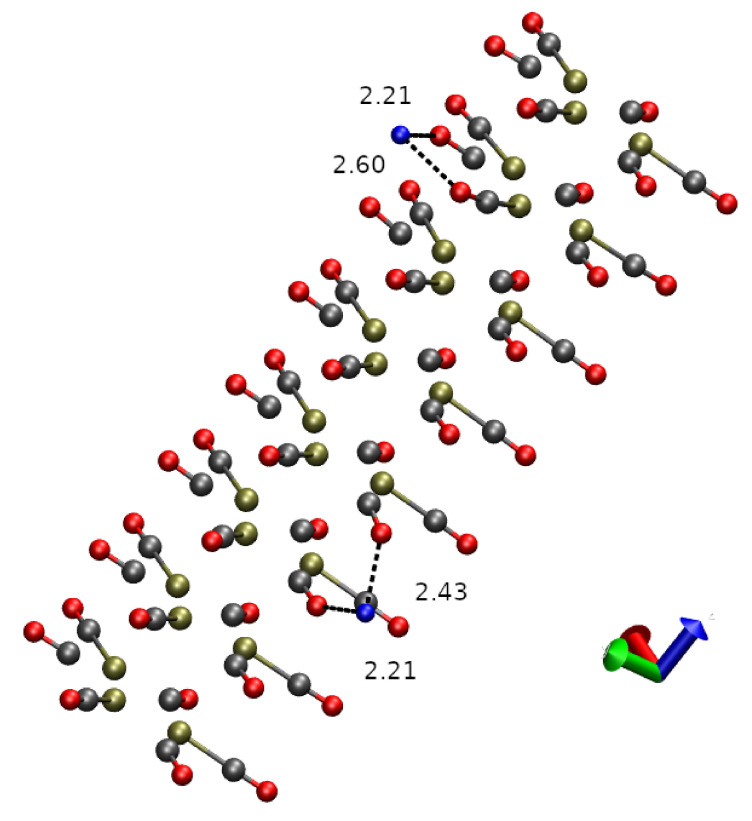
Final configuration (conf. 150 in Figure 6). Na atoms are displayed as blue spheres. The shortest distances between Na and O atoms in the nano-wire are displayed. For clarity, bonds are drawn when distance between atoms in the pair are shorter than 1.6 Å.

As for the extended nano-wire, we computed the DIs for several snapshots along with the variable-cell energy minimization in the presence of mobile cations like Na^+^. When Na atoms are far from the nano-wire, the charge integrated over the Na atomic basins is close to 1, showing that in this case the charge separation is correctly modeled. When the Na cations become free to move, they rapidly approach the negatively charged nano-wire. This process is displayed in Figure 6, where the evolution of some geometrical parameters is reported along with the variable-cell energy minimization. The first 50 points of the energy minimization were performed with Na atoms fixed in space. The minimal distance between the Na atoms and the nano-wire (O atoms) is larger than 5 Å. At the beginning (conf. zero), the structure of the nano-wire is that of the minimal energy configuration obtained with cell charge −2 and with fixed cell sides. Keeping the Na position fixed while allowing the cell side relaxation, the reduction of nano-wire periodicity is the major structural change (panels A and C, black curve): all the Pt–Pt inter-monomer distances regularly adapt to the neutrality of the cell. The release of constraints acting on Na atoms (point 50) allows Na to reach the nano-wire and, when the minimal Na-O distance achieves 5 Å, the energy starts decreasing rapidly. After the first encounter between Na and O atoms (point 80), a slow settling of interactions between Na and O atoms occurs. The final collected configuration (point 150) is displayed in Figure 7. The corresponding evolution of the Pt–Pt inter-monomer minimal distances are displayed in Figure 6C. This plot shows that the interactions between Na and the nano-wire significantly affect the distance between the stack of monomer Pt_3_ planes. The regular inter-monomer distance displayed by configuration 50 is altered and slowly settled to a range of values 3.1 ÷ 3.25 Å (configuration 150). This effect clearly shows that the approach of small cations towards the nano-wire modifies the regular inter-monomer distance, introducing a little structural defect. We are now in the position to monitor the effect of such defects on the electron sharing between Pt_3_ stacks.

**Figure 8:**
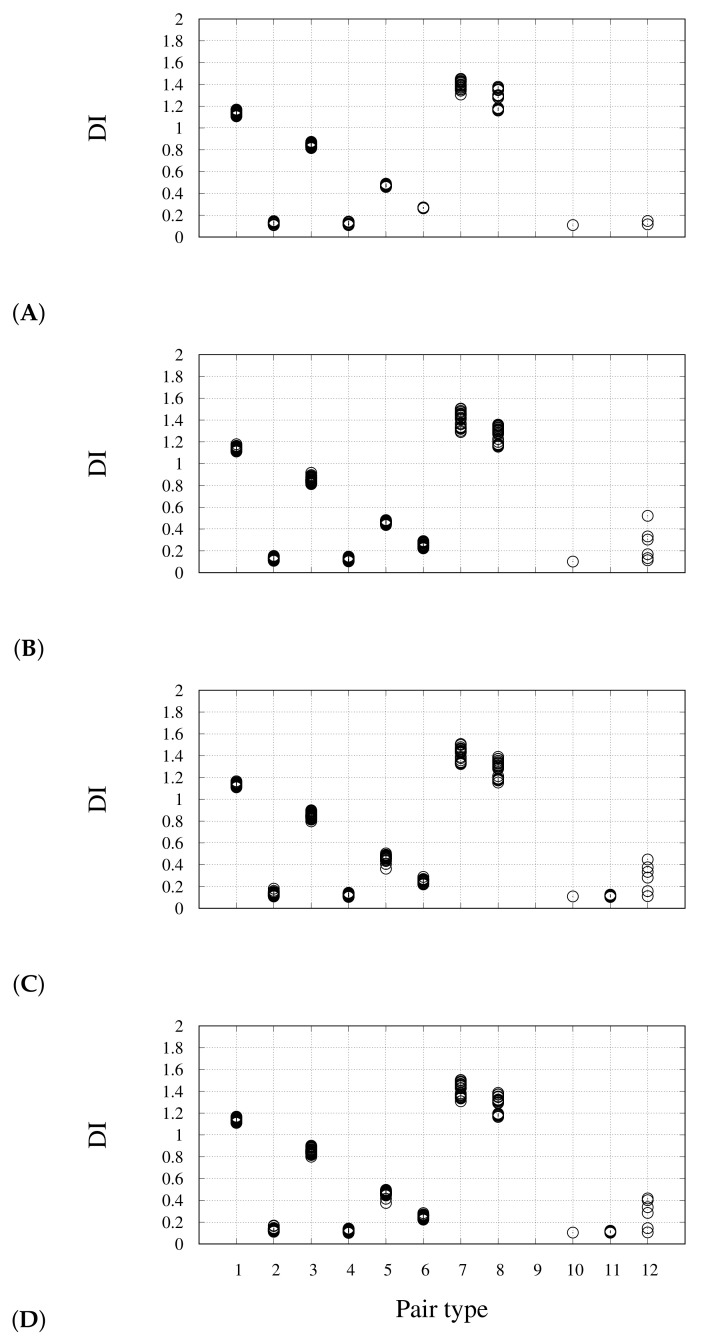
DI (*y* axis) for different atom pairs (see list below and Figure 2) along with the approach of Na towards the molecule: (**A**) initial configuration (conf. 30 in Figure 6); (**B**) first-encounter configuration (conf. 100 in Figure 6); (**C**) minimal energy configuration (conf. 148 in Figure 6); (**D**) settled configuration (conf. 150 in Figure 6 and Figure 7). List of atom pairs: 1—Pt1-Ct; 2—Pt1-Ot; 3—Pt1-Cb; 4—Pt1-Ob; 5—Pt1-Pt1; 6—Pt1-Pt16; 7—Ct-Ot; 8—Cb-Ob; 10—different from above, but intra-monomer; 11—as in 10, but inter-monomer; 12—Na-molecule.

The pattern of DIs for different pair types is displayed in Figure 8 for several snapshots, along with the variable cell relaxation. The selected snapshots are indicated as circles in Figure 6A. In panel A, corresponding to the negatively charged nano-wire separated from the two Na cations, we notice that no DIs are measured for pair types 10 and 11 (see Figure 5C). This confirms the requirement of avoiding the assignment of space points to atoms in regions of space where electron density is low and flat. This can be easily done with counterions. We notice here that DI(Pt–Ct) is lower in the nano-wire (1.2) than in the monomer (1.5) (see Table 1 and Figure 5A). When Na atoms become close to CO ligands, a significant electron sharing appears involving Na basins (panel B), even if the charge assigned to Na atoms is still approximately 1 (0.95) and this value is maintained during the following Na settling around the CO ligand atoms. The charges integrated over atomic basins do not change appreciably, especially when summed over monomers (data not shown). This indicates that the charge distribution is not affected by the interactions between the negatively charged wire and the positively charged counterions. The largest DI occurs for Na–Ob pairs (up to 0.6, panel B), while the largest value for Na–Ot pair is 0.3. The electrons shared between Na and Ob atoms are extracted by Pt–Pt pairs (pair types 5 and 6) where the perturbation of symmetry becomes evident after the first Na-O encounter (panels C-D). However, the effect on electron sharing within the nano-wire of the Na-O interactions at the periphery is extremely small, showing that the CO insulating effect is particularly strong.

## 4. Materials and Methods

### 4.1. Ground-State Electron Density and Energy Minimization

The ground-state electron density was computed by using Vanderbilt ultra-soft pseudo-potentials [27] and the PBE exchange-correlation functional [28]. Electronic wave function was expanded in plane waves up to an energy cut-off $E_{w}=30$ Ry, while a cut-off $E_{d}=250$ Ry was used for the expansion of the augmented charge density in the proximity of the atoms, as required in the ultra-soft pseudo-potential scheme (see Section 2.2). Periodic boundary conditions were applied in the three direction of space. The Pt_3_ plane was always initially parallel to the cell xy plane and cell side $L_{x}=L_{y}=$ 2 nm. $L_{z}$ was initially set to 2.48 nm. All calculations were performed under spin-restricted conditions, i.e., with each Kohn–Sham orbital filled with two electrons of opposite spin. A Gaussian spreading of KS occupation was used to prevent problems in self-consistency convergence. The energy was minimized using a tolerance of $10^{-6}$ Ry. The energy and atomic forces are corrected for the effects of cells with net charge different from zero [29,30].

DFT–D3 dispersion interactions [31] were included for energy and force calculations in order to correct for the lack of such interactions in the PBE approximation to DFT. The energy minimization was performed with the Broyden–Fletcher–Goldfarb–Shanno algorithm until all force components were smaller than $10^{-4}$ Ry/bohr. In the variable-cell energy minimization, only $L_{z}$ was allowed to relax, while $L_{x}$ and $L_{y}$ were kept fixed to 2 nm. The number of steps required by energy minimization did not exceed 150.

### 4.2. Including All Electrons

The frozen-core contribution of every atom is added to the valence electron density in the real-space representation. This is needed because, in a pseudo-potential framework, the charge density, which is also pseudized, is very small in the region close to the nucleus and may cause the atomic basin identification algorithms of Section 4.3 to fail. Since we do not need an accurate all-electron charge density reconstruction, we have resorted to a simple method, implemented as a new option of the QE post-processing tools. The charge of each frozen core is built according to the Zener–Slater approximation of isolated atoms [32,33]. The radial part of each one-electron effective state is:

(14) $$R\left(r\right)=R_{0}r^{\left(n^{*}-1\right)}\exp\left[-\left(Z-s\right)\frac{r}{n^{*}a_{0}}\right]\;\;,$$

where *r* is the distance from the nucleus, $a_{0}$ is the Bohr radius, *Z* is the atomic number, *s* and $n^{*}$ are screening parameters, and $R_{0}$ is a normalization constant. The parameters are tabulated in textbooks [34]. The charge assigned to each grid point in the dense real-space representation of valence electron density is augmented by the frozen core charge, averaged over a sub-grid of 16 × 16 × 16 regularly spaced points in a cube surrounding each grid point. Only points within the core radius of each pseudo-potential (in the range of 3 bohr) contribute. This procedure allows a smooth spreading of core charge among the cells in the dense-grid representation of electron density. The correctness of integrals of the all-electron density in all cases was checked to be within 1%.

### 4.3. Atomic Basins Identification

Approximated all-electron densities were analyzed with the approach known as quantum theory of atoms in molecules [16,17]. The method assigns the set of points in space confined within the surfaces of zero flux of the electron density gradient to the atom within the respective surface. This analysis was performed with the algorithm initially proposed by Sanville et al. [18]. The version 1.03 of the code was used in this work. Once the atomic basins Ω were identified, the integral for each DI in Equation (3) was computed simply by summing over the set of elementary cubic volumes identified by each of the atomic basin Ω. Therefore, the implementation of Equation (3) requires the simple collection of 3D grids of the valence electron density and of the set of KS molecular orbitals representing it, in whatever representation.

The electron density was collected in real space on the dense 3-D grid used in PW calculations with ultra-soft pseudo-potentials. The final 3-D representation of electron density has a finite-element side that depends upon the energy cut-off $E_{d}$. For the $E_{d}=250$ Ry cut-off we used, the finite-element side is about 10 p.m. By increasing $E_{d}$, a finer real-space grid is obtained. The convergence of Equation (3) can thus be assessed by increasing the resolution of electron density. In all cases, the valence electron density was complemented with the core electron density by the procedure described above.

### 4.4. Parallelization

The calculation of DIs requires a large amount of memory, since the real-space representation of all valence KS states must be kept in memory for the calculation of overlap integrals in the different atomic basins. This memory requirement limits the size of systems for which the calculations of DIs is possible, even with high-performance computing hardware. With the parallelization scheme of QE, we could perform the calculation of DIs for 120 atoms, without storing the KS states on disk, a strong limitation of our previous post-processing code [13].

QE has several ways to distribute tasks in parallel architectures. Products, scalar products, sums over the $\vec{G}$ and $\vec{r}$ grids, as well as three-dimensional FFTs, can be easily parallelized using the available “plane-wave” parallelization of QE.

For the calculation of DIs, it is convenient to resort to “band parallelization”, where groups of KS states are managed by independent computational tasks (the index *i* of KS states $\psi_{i}$ is distributed). In order to exploit band parallelization without replicating arrays over all processors, we resort to the following algorithm.

The *N* processors are divided into $n_{b}$ “band groups” of $N/n_{b}$ processors each. Initially, each band group contains $M_{b}=M/n_{b}$ KS states $\psi_{i}$ (from $i=\left(i_{b}-1\right)M_{b}+1$ to $i=i_{b}M_{b}$ for band group $i_{b}$). The $\vec{r}$ and $\vec{G}$ components of all needed arrays are distributed across the processors inside each band group.

$\rho_{i,j}^{us}$ is first computed for $i,j=\left(i_{b}-1\right)M_{b}+1,\ldots ,i_{b}M_{b}$. The FFTs, products and scalar products are parallelized inside each band group and the various contributions to the integrals are summed.

Band group $i_{b}$ then sends a copy of $\psi_{i}$ to band group $i_{b}-1$, receives the copy sent from processor $i_{b}+1$ (odd numbered processes send first, receive later; even-numbered processes receive first, send later).

It is now possible to compute $\rho_{i,j}^{us}$ for $i=\left(i_{b}-1\right)M_{b}+1,\ldots ,i_{b}M_{b}$ and $j=i_{b}M_{b}+1,\ldots ,\left(i_{b}+1\right)M_{b}$. The procedure is iterated until each band group has a complete slice of $\rho_{i,j}^{us}$ for all values of *j*. The partial results for each band group are then collected to yield the final result.

## 5. Conclusions

The concept of covalent bond is here fully recovered by properly analyzing density-functional theory calculations performed in a basis set that does not depend upon atomic positions and type. This is the case of plane waves, frequently used for condensed phases with periodic boundary conditions. Delocalization indices, measuring electron sharing between any pair of atoms, can thus be computed in extended systems made of more than one hundred of atoms. Our method takes profit of the short-range character of augmentation charge and of the double-grid method to compute the required overlap integrals in a quick and effective way. The new method to compute delocalization index is applied to a Pt nano-wire isolated by CO molecules, where different extents of electron sharing are observed, like charge polarization of CO ligands and metal–metal bonding.

The algorithm is fast, reliable, and is provided as a routine tool within the Quantum ESPRESSO open-source package, to better understand electron distribution in complex materials.

## Figures and Tables

**Table 1:** DIs for types of atomic pairs as indicated in Figure 2. Pt1 indicates Pt of the displayed monomer, while Pt16 indicates the closest Pt atom of the nearby monomer in the stack. These values are obtained with no counterions, using the minimal energy configurations obtained with total charge q=−2 in all cases.

Pair	Type	*n*			
		1	2	4	8
Pt1-Ct	1	1.5	1.4	1.3	1.3
Pt1-Ot	2	0.25	0.24	0.23	0.22
Pt1-Cb	3	0.88	0.85	0.81	0.78
Pt1-Ob	4	0.16	0.16	0.15	0.14
Pt1-Pt1	5	0.55	0.48	0.47	0.46
Pt1-Pt16	6	-	0.35	0.34	0.28
Ct-Ot	7	1.6	1.6	1.6	1.6
Cb-Ob	8	1.5	1.5	1.5	1.6

## Data Availability

Not applicable.

## Sample Availability

Samples of the compounds are not available from the authors.

## References

[1] Parr R.G., Yang W. (1989). Density Functional Theory of Atoms and Molecules.

[2] Car R., Parrinello M. (1985). Unified Approach for Molecular Dynamics and Density-Functional Theory. Phys. Rev. Lett..

[3] Outeiral C., Vincent M.A., Martín Pendás Á., Popelier P.L.A. (2018). Revitalizing the concept of bond order through delocalization measures in real space. Chem. Sci..

[4] Fradera X., Poater J., Simon S., Duran M., Solà M. (2002). Electron-pairing analysis from localization and delocalization indices in the framework of the atoms-in-molecules theory. Theor. Chem. Acc..

[5] Matito E., Poater J., Solà M., Duran M., Salvador P. (2005). Comparison of the AIM delocalization index and the Mayer and fuzzy atom bond orders. J. Phys. Chem. A.

[6] Bakó I., Stirling A., Seitsonen A., Mayer I. (2013). Extracting chemical information from plane wave calculations by a 3D fuzzy atoms analysis. Chem. Phys. Lett..

[7] Szczepanik D.W., Andrzejak M., Dominikowska J., Pawełek B., Krygowski T.M., Szatylowicz H., Solá M. (2017). The electron density of delocalized bonds (EDDB) applied for quantifying aromaticity. Phys. Chem. Chem. Phys..

[8] Wannier G.H. (1937). The Structure of Electronic Excitation Levels in Insulating Crystals. Phys. Rev..

[9] Otero-de-la Roza A., Martín Pendás Á., Johnson E.R. (2018). Quantitative Electron Delocalization in Solids from Maximally Localized Wannier Functions. J. Chem. Theory Comput..

[10] Golub P., Baranov A.I. (2016). Domain overlap matrices from plane-wave-based methods of electronic structure calculation. J. Chem. Phys..

[11] Kohout M. (2019). DGrid.

[12] Otero-de-la Roza A., Blanco M., Martín Pendás A., Luaña V. (2009). Critic: A new program for the topological analysis of solid-state electron densities. Comp. Phys. Commun..

[13] La Penna G., Furlan S., Solá M. (2011). Measuring Electron Sharing Between Atoms in First-Principle Simulations. Theor. Chem. Acc..

[14] Giannozzi P., Baroni S., Bonini N., Calandra M., Car R., Cavazzoni C., Ceresoli D., Chiarotti G.L., Cococcioni M., Dabo I. (2009). QUANTUM ESPRESSO: A Modular and Open-Source Software Project for Quantum Simulations of Materials. J. Phys. Condens. Matter.

[15] Giannozzi P., Andreussi O., Brumme T., Bunau O., Buongiorno Nardelli M., Calandra M., Car R., Cavazzoni C., Ceresoli D., Cococcioni M. (2017). Advanced capabilities for materials modelling with Quantum ESPRESSO. J. Phys. Condens. Matter.

[16] Bader R.F.W., Stephens M.E. (1975). Spatial localization of the electronic pair and number distributions in molecules. J. Am. Chem. Soc..

[17] Bader R.F.W. (1994). Atoms in Molecules—A Quantum Theory.

[18] Sanville E., Kenny S.D., Smith R., Henkelman G. (2007). Improved grid-based algorithm for Bader charge allocation. J. Comput. Chem..

[19] Tang W., Sanville E., Henkelman G. (2009). A grid-based Bader analysis algorithm without lattice bias. J. Phys. Condens. Matter.

[20] Giannozzi P., De Angelis F., Car R. (2004). First-Princple Molecular Dynamics with Ultrasoft Pseudopotentials: Parallel Implementation and Application to Extended Bioinorganic Systems. J. Chem. Phys..

[21] Macchi P., Sironi A. (2003). Chemical bonding in transition metal carbonyl clusters: Complementary analysis of theoretical and experimental electron densities. Coord. Chem. Rev..

[22] Racioppi S., Della Pergola R., Colombo V., Sironi A., Macchi P. (2018). Electron Density Analysis of Metal Clusters with Semi-Interstitial Main Group Atoms. Chemical Bonding in [Co_6_X(CO)_16_]^−^ Species. J. Phys. Chem. A.

[23] Ciabatti I., Femoni C., Iapalucci M.C., Longoni G., Zacchini S. (2014). Platinum Carbonyl Clusters Chemistry: Four Decades of Challenging Nanoscience. J. Clust. Sci..

[24] Berti B., Femoni C., Iapalucci M.C., Ruggieri S., Zacchini S. (2018). Functionalization, Modification, and Transformation of Platinum Chini Clusters. Eur. J. Inorg. Chem..

[25] Femoni C., Kaswalder F., Iapalucci M.C., Longoni G., Mehlstäubl M., Zacchini S., Ceriotti A. (2006). Synthesis and crystal structure of [NBu4]2[Pt24(CO)48]: An infinite 1d stack of Pt3(CO)6 units morphologically resembling a CO-insulated platinum cable. Angew. Chemie Int. Ed. Engl..

[26] Humphrey W., Dalke A., Schulten K. (1996). VMD visual molecular dynamics. J. Molec. Graph..

[27] Vanderbilt D. (1990). Soft Self-Consistent Pseudopotentials in a Generalized Eigenvalue Formalism. Phys. Rev. B.

[28] Perdew J.P., Burke K., Ernzerhof M. (1996). Generalized Gradient Approximation Made Simple. Phys. Rev. Lett..

[29] Makov G., Payne M.C. (1995). Periodic Boundary Conditions in *Ab Initio* Calculations. Phys. Rev. B.

[30] Martyna G.J., Tuckerman M.E. (1999). A Reciprocal Space Based Method for Treating Long Range Interactions in Ab Initio and Force-Field-Based Calculations in Clusters. J. Chem. Phys..

[31] Grimme S., Antony J., Ehrlich S., Krieg H. (2010). A Consistent and Accurate Ab Initio Parametrization of Density Functional Dispersion Correction (DFT-D) for the 94 Elements H-Pu. J. Chem. Phys..

[32] Zener C. (1930). Analytic Atomic Wave Functions. Phys. Rev..

[33] Slater J.C. (1930). Atomic Shielding Constants. Phys. Rev..

[34] Eyring H., Walter J., Kimball G. (1944). Quantum Chemistry.

